# Association between *methylenetetrahydrofolate reductase* tagging polymorphisms and susceptibility of hepatocellular carcinoma: a case–control study

**DOI:** 10.1042/BSR20192517

**Published:** 2019-11-12

**Authors:** Sheng Zhang, Jing Lin, Jiakai Jiang, Yu Chen, Weifeng Tang, Longgen Liu

**Affiliations:** 1 Department of General Surgery, Changzhou No. 3 People’s Hospital, Changzhou, Jiangsu Province, China; 2 Department of Medical Oncology, Fujian Cancer Hospital and Fujian Medical University Cancer Hospital, Fuzhou, Fujian Province, China; 3 Department of Cardiothoracic Surgery, Affiliated People’s Hospital of Jiangsu University, Zhenjiang, Jiangsu Province, China; 4 Department of Liver Disease, Changzhou No. 3 People’s Hospital, Changzhou, Jiangsu Province, China

**Keywords:** Hepatocellular carcinoma, MTHFR, Polymorphism, Susceptibility, Tagging

## Abstract

Polymorphisms in one-carbon metabolism genes may influence the susceptibility to hepatocellular carcinoma (HCC). In the present study, we studied methylenetetrahydrofolate reductase (*MTHFR*) tagging polymorphisms in 584 HCC cases and 923 controls. Polymerase chain reaction was harnessed to detect *MTHFR* genotype. Overall, our results showed that genotype distribution of *MTHFR* rs4846048 and rs4845882 polymorphisms was not different between HCC patients and controls. *MTHFR* rs9651118 and rs1801133 loci were protective factors for HCC (rs9651118: CT vs. TT: adjusted odds ratio (OR) = 0.67, 95% confidence interval (CI): 0.49–0.90, *P*=0.008 and TC/CC vs. TT: adjusted OR = 0.70, 95% CI: 0.53–0.93, *P*=0.015; rs1801133: GA vs. GG: adjusted OR = 0.72, 95% CI: 0.54–0.97, *P*=0.031, AA/GA vs. GG: adjusted OR = 0.76, 95% CI: 0.57–0.99, *P*=0.045). However, *MTHFR* rs3753584 locus was a candidate for susceptibility to HCC (CT vs. TT: adjusted OR = 1.67, 95% CI: 1.20–2.32, *P*=0.003 and TC/CC vs. TT: adjusted OR = 1.59, 95% CI: 1.15–2.20, *P*=0.005). Results of haplotype analysis suggested that *MTHFR* G_rs1801133_T_rs3753584_G_rs4845882_A_rs4846048_T_rs9651118_ was associated with the risk of HCC (OR = 1.55, 95% CI: 1.16–2.07, *P***=**0.003). The power of our study also confirmed these associations (the value of power >0.80). In summary, our findings suggested that *MTHFR* rs3753584, rs9651118 and rs1801133 polymorphisms may affect the risk of HCC in Chinese Han population. In future, our findings should be further validated in additional case–control studies.

## Introduction

In 2015, liver cancer (LC) ranked the third most frequent type of malignancy in males and the sixth most frequent type in females, approximately 343700 and 122300 cases occuring in China, respectively [1]. The total LC-related deaths are the third most frequent type of malignancy [1,2]; however, the etiology of LC remains unclear. Susceptibility factors [e.g. hepatitis B virus (HBV), age, obesity, type 2 diabetes, consumption of food contaminated with aflatoxin, nonalcoholic fatty liver disease, heavy drinking related cirrhosis, and tobacco use] may be implicated in the etiology of LC [3–7]. Accumulating evidences suggested that besides these mentioned people’s lifestyle and environmental factors, some genetic predispositions might also contribute to development of LC.

Folic acid is important for DNA synthesis and methylation, mitosis and controlling related gene expression. Recently, epidemiological investigations showed that sufficient fruits and vegetables intake may be a protective factor for carcinogenesis [8–10]. It is thought that these potential protective roles of diet attribute to the high level intake of folic acid. Methylenetetrahydrofolate reductase (MTHFR) plays an important role in catalyzing the transition of 5,10-methylenetetrahydrofolate to 5-methyltetrahydrofolate (5-MTHF), which is a main plasm form of folate. And 5-MTHF is implicated in a conversion procedure of homocysteine (Hcy) into methionine (Met) and the methylation of DNA. Considering the vital role of MTHFR, variants in *MTHFR* gene may influence the development of cancer.

In humans, MTHFR protein is coded by the *MTHFR* gene which is located on chromosome 1p36.3. *MTHFR* single nucleotide polymorphisms (SNPs) may be a potential biomarker of cancer. In addition, Jiao et al. [11] reported that hepatocellular carcinoma (HCC) patients with HBV-infection carried *MTHFR* rs1801133 AA genotype and A allele may have a better prognosis than those who carried *MTHFR* rs1801133 GG genotype and G allele. Recently, some studies investigated a potential association of *MTHFR* loci with HCC susceptibility [11–15]; however, due to the limited sample sizes, the observations might be conflicting. Therefore, in the present study, we included 1507 subjects to perform a case–control study to extensively explore the relationship between *MTHFR* tagging SNPs and the risk of HCC.

## Materials and methods

### Study population

Our study recruited 584 consecutive HCC cases from two Clinical Medical College of Fujian Medical University (Fuzhou City, China) during 2002–2016. Two doctors confirmed HCC diagnosis by pathology. The criterion of Barcelona Clinic Liver Cancer (BCLC) was used to determine the stage of HCC [16,17]. In addition, 923 Chinese people without any cancer history were included as controls. We matched HCC patients and controls by region (Eastern China), age and sex. Every subject was notified purpose of the study. All participants provided written informed consent. A questionnaire regarding age, sex, smoking and alcohol status was used to collect the corresponding information. The protocol of this investigation was approved by Ethics Committee of Fujian Medical University. In the present study, the principles of Declaration of Helsinki was conformed. Chronic HBV infection were determined by using hepatitis B surface antigen enzyme-linked immunosorbent assay Kit (InTec, Xiamen, China).

### SNPs selection and genotyping

*MTHFR* tagging SNPs were selected through Haploview software, which are described in our previous case–control studies [18,19].

EDTA-anticoagulated blood was donated and collected. According to the standard experimental protocol, genomic DNA was obtained by using a DNA Kit (Promega, Madison, U.S.A.). A SNPscan Kit (Genesky Biotechnologies Inc., Shanghai, China) was used to determine the variants of *MTHFR* SNPs as described in our case–control studies [20]. At this stage, 60 DNA samples were selected and re-analyzed for quality control. Finally, the genotype frequencies were not changed.

### Statistical analysis

In the present study, *χ^2^* test or Fisher’s exact test was harnessed to analyze the differences in sex, chronic HBV infection, age, smoking, drinking and the genotype frequencies of HCC patients compared with controls. In addition, age was expressed as means ± standard deviation (SD). The difference in age between two groups was determined by Student’s *t* test. With an internet Hardy–Weinberg equilibrium (HWE) test (http://ihg.gsf.de/cgi-bin/hw/hwa1.pl), genotype frequencies among controls was used to assess the HWE status [21,22,23,24]. The correlation between *MTHFR* SNPs and HCC risk was evaluated by odds ratios (ORs) and 95% confidence intervals (CIs). Logistic regression analysis was used to determine the associations between *MTHFR* polymorphisms and the risk of HCC by adjusting sex, chronic HBV infection, age, smoking and drinking. A two-tailed *P*<0.05 was considered as significant. We used SHESIS online program (http://analysis.bio-x.cn/myAnalysis.php) to construct *MTHFR* haplotypes [25]. SAS 9.4 version statistical software (SAS Institute, Cary, NC) was used to analyze data. The power of the present study was determined by a power calculating software (http://biostat.mc.vanderbilt.edu/twiki/bin/view/Main/PowerSampleSize) (α = 0.05) [26].

## Results

### Characteristics

As described in Table 1, 1507 participants (584 patients and 923 controls) were included in the present study. Male:female ratio of the HCC patients was ∼9:1 (89.90% and 10.10%), controls were recruited in the similar proportion (90.47% males and 9.53% females) to match the distribution of sex and average age (HCC cases = 53.17, SD ± 11.76 years; controls = 53.72, SD ± 9.97 years; *P*=0.327). There were 210 (35.96%) smokers among HCC group and 327 (35.43%) among non-cancer controls, while non-smokers were 374 (64.04%) in HCC cases and 596 (64.57%) in the controls. Drinking and chronic HBV infection ratio in HCC patients was higher than that of controls (29.11 vs. 16.03% and 70.55 vs. 9.21%, respectively). The BCLC stage of HCC was summarized in Table 1. The data of SNPs in *MTHFR* gene were listed in Table 2.

**Table 1:** Distribution of selected demographic variables and risk factors in HCC cases and controls

Variable	Cases (*n*=584)	Controls (*n*=923)	*P*^1^
	*n* (%)	*n* (%)	
Age (years)	53.17 (±11.76)	53.72 (±9.97)	0.327
Age (years)			0.358
<53	264 (45.21)	395 (42.80)	
≥53	320 (54.79)	528 (57.20)	
Sex			0.717
Male	525 (89.90)	835 (90.47)	
Female	59 (10.10)	88 (9.53)	
Smoking status			0.834
Never	374 (64.04)	596 (64.57)	
Ever	210 (35.96)	327 (35.43)	
Alcohol use			<0.001
Never	414 (70.89)	775 (83.97)	
Ever	170 (29.11)	148 (16.03)	
Chronic HBV infection			<0.001
Yes	412 (70.55)	85 (9.21)	
No	172 (29.45)	838 (90.79)	
BCLC classification			
A	392 (67.12)		
B	175 (29.97)		
C	17 (2.91)		

Bold values are statistically significant (*P*<0.05).

1 Two-sided *χ*^2^ test and Student’s *t* test.

**Table 2:** Primary information for *MTHFR* polymorphisms

Genotyped SNPs	rs3753584 T>C	rs4846048 A>G	rs4845882 G>A	rs1801133 G>A	rs9651118 T>C
Chromosome	1	1	1	1	1
Location (NCBI Build 37)	11864586	11846252	11843167	11856378	11862214
Function	NearGene-5	Intron	Intron	Missense	Intron
Regulome DB scores (http://www.regulomedb.org/)	4	3a	1f	4	5
Transcription factor binding site (http://snpinfo.niehs.nih.gov/snpinfo/snpfunc.htm)	Y	-	-	-	Y
MiRNA (miRanda)	-	Y	-	-	-
MAF^1^ for Chinese in database	0.093	0.105	0.198	0.439	0.382
MAF in our controls (*n*=923)	0.111	0.095	0.216	0.354	0.378
*P*-value for HWE^2^ test in our controls	0.814	0.029	0.437	0.074	0.021
Genotyping method	SNPscan	SNPscan	SNPscan	SNPscan	SNPscan
% Genotyping value	99.27%	99.27%	99.27%	99.27%	99.27%

1 MAF, minor allele frequency.

2 HWE.

### Relationship of MTHFR polymorphisms with HCC patients

Table 3 summarizes the *MTHFR* genotype frequencies in HCC patients and control groups. Overall, we found that *MTHFR* rs4846048 and rs4845882 genotype distribution were not statistically significant between two groups.

**Table 3:** Logistic regression analyses of associations between *MTHFR* rs3753584 T>C, rs4845882 G>A, rs1801133 G>A, rs4846048 A>G and rs9651118 T>C polymorphisms and the risk of HCC

Genotype	Cases (*n*=584)		Controls (*n*=923)		Crude OR (95% CI)	*P*	Adjusted OR^1^ (95% CI)	*P*
	*n*	%	*n*	%				
*MTHFR* rs1801133 G>A								
GG	299	52.00	372	40.39	1.00		1.00	
GA	227	39.48	446	48.43	**0.63 (0.51–0.79)**	**<0.001**	**0.72 (0.54–0.97)**	**0.031**
AA	49	8.52	103	11.18	**0.59 (0.41–0.86)**	**0.006**	0.89 (0.56–1.42)	0.625
GA + AA	276	48.00	549	59.48	**0.63 (0.51–0.77)**	**<0.001**	**0.76 (0.57–0.99)**	**0.045**
GG+ GA	526	91.48	818	88.82	1.00		1.00	
AA	49	8.52	103	11.18	0.74 (0.52–1.06)	0.098	1.05 (0.67–1.64)	0.842
A allele	325	28.26	652	35.40				
*MTHFR* rs3753584 T>C								
TT	431	74.96	729	79.15	1.00		1.00	
CT	139	24.17	180	19.54	**1.31 (1.02–1.68)**	**0.037**	**1.67 (1.20–2.32)**	**0.003**
CC	5	0.87	12	1.30	0.71 (0.25–2.01)	0.514	0.64 (0.16–2.57)	0.530
CT+CC	144	25.04	192	20.85	1.27 (0.99–1.62)	0.059	**1.59 (1.15–2.20)**	**0.005**
TT+CT	570	99.13	909	98.70	1.00		1.00	
CC	5	0.87	12	1.30	0.67 (0.23–1.90)	0.445	0.58 (0.15–2.29)	0.434
C allele	149	12.96	204	11.07				
*MTHFR* rs4845882 G>A								
GG	329	57.22	562	61.02	1.00		1.00	
GA	222	38.61	320	34.74	1.19 (0.95–1.48)	0.128	1.26 (0.94–1.67)	0.121
AA	24	4.17	39	4.23	1.05 (0.62–1.78)	0.853	1.18 (0.59–2.36)	0.650
GA+AA	246	42.78	359	38.98	1.17 (0.95–1.45)	0.145	1.25 (0.94–1.65)	0.120
GG+GA	551	95.83	882	95.77	1.00		1.00	
AA	24	4.17	39	4.23	0.99 (0.59–1.66)	0.955	1.08 (0.54–2.15)	0.831
A allele	270	23.48	398	21.61				
*MTHFR* rs4846048 A>G								
AA	465	80.87	760	82.52	1.00		1.00	
AG	107	18.61	147	15.96	1.19 (0.90–1.57)	0.215	1.17 (0.82–1.68)	0.395
GG	3	0.52	14	1.52	0.35 (0.10–1.23)	0.101	0.26 (0.06–1.23)	0.090
AG+GG	110	19.13	161	17.48	1.12 (0.85–1.46)	0.421	1.08 (0.76–1.54)	0.668
AA+AG	572	99.48	907	98.49	1.00		1.00	
GG	3	0.52	14	1.52	0.34 (0.10–1.19)	0.091	0.25 (0.05–1.20)	0.083
G allele	113	9.82	175	9.50				
*MTHFR* rs9651118 T>C								
TT	216	37.57	340	36.92	1.00		1.00	
TC	267	46.43	466	50.60	0.90 (0.72–1.13)	0.373	**0.67 (0.49–0.90)**	**0.008**
CC	92	16.00	115	12.49	1.26 (0.91–1.74)	0.162	0.85 (0.55–1.31)	0.458
TC+CC	359	62.43	581	63.08	0.97 (0.78–1.21)	0.800	**0.70 (0.53–0.93)**	**0.015**
TT+TC	483	84.00	806	87.51	1.00		1.00	
CC	92	16.00	115	12.49	1.34 (0.99–1.80)	0.056	1.07 (0.72–1.59)	0.758
C allele	451	39.22	696	37.79				

1 Adjusted for age, sex, smoking, status of chronic HBV infection and drinking.

Bold values are statistically significant (*P*<0.05).

Compared with rs1801133 GG genotype frequency, we found that *MTHFR* rs1801133 GA genotype significantly decreased the risk of HCC (*P*<0.001). When rs1801133 GG frequency was used as reference, there was a difference in *MTHFR* rs1801133 AA and AA/GA genotype frequency between two groups (*P*=0.006 and *P*<0.001, respectively). Adjustment for sex, chronic HBV infection, age, smoking and drinking, the significant decreased risk also re-appeared in two genetic models (GA vs. GG: adjusted *P*=0.031, AA/GA vs. GG: adjusted *P*=0.045, respectively).

We also found *MTHFR* rs3753584 had an increased susceptibility to HCC (CT vs. TT: *P*=0.037). Adjustments for sex, chronic HBV infection, age, smoking and drinking, the findings were more significant (CT vs. TT: adjusted *P*=0.003 and TC/CC vs. TT: adjusted *P*=0.005).

In crude comparison, we did not find any relationship between *MTHFR* rs9651118 and HCC susceptibility. However, when adjusted for sex, chronic HBV infection, age, smoking and drinking, a statistically decreased risk of HCC was identified in two genetic model (CT vs. TT: adjusted *P*=0.008 and TC/CC vs. TT: adjusted *P*=0.015).

### Association of MTHFR polymorphisms with HCC in a stratification analysis

Table 4 showed the *MTHFR* genotype frequencies in the subgroup analyses by the status of chronic HBV infection. We found that *MTHFR* rs1801133 and rs9651118 polymorphisms were associated with the decreased risk of HCC in no chronic HBV infection subgroup (rs1801133: GA vs. GG: adjusted *P*=0.001 and GA/AA vs. GG: adjusted *P*=0.001; and rs9651118: CT vs. TT: adjusted *P*=0.021 and TC/CC vs. TT: adjusted *P*=0.037). However, we identified that *MTHFR* rs1801133 polymorphism was associated with HCC risk in chronic HBV infection subgroup (rs1801133: AA vs. GG: adjusted *P*=0.035 and GA/AA vs. GG: adjusted *P*=0.035). Additionally, the association of *MTHFR* rs3753584 and rs4845882 polymorphism with the risk of HCC was also found (rs3753584: CT vs. TT: adjusted *P*=0.003 and TC/CC vs. TT: adjusted *P*=0.005; rs4845882: GA vs. GG: adjusted *P*=0.022 and GA/AA vs. GG: adjusted *P*=0.021).

**Table 4:** Stratified analyses between *MTHFR* polymorphisms and HCC risk by status of chronic HBV infection

Genotype	Chronic HBV infection (Yes)				Adjusted OR^1^	*P*^1^	Chronic HBV infection (No)				Adjusted OR^1^	*P*^1^
	Case		Control				Case		Control			
	*n*	%	*n*	%			%	*n*	*n*	%		
*MTHFR* rs1801133 G>A												
GG	210	51.98	51	60.00	1.00		89	52.05	321	38.40	1.00	
GA	163	40.35	32	37.65	1.53 (0.91–2.79)	0.111	64	37.43	414	49.52	**0.53 (0.37–0.76)**	**0.001**
AA	31	7.67	2	2.35	**5.06 (1.12–22.88)**	**0.035**	18	10.53	101	12.08	0.63 (0.36–1.10)	0.104
GA + AA	194	48.02	34	40.00	** 1.73 (1.04–2.87)**	**0.035**	82	47.95	515	61.60	**0.55 (0.39–0.77)**	**0.001**
GG+ GA	373	92.33	83	97.65	1.00		153	89.47	735	87.92	1.00	
AA	31	7.67	2	2.35	4.20 (0.95–18.67)	0.059	18	10.53	101	12.08	0.86 (0.50–1.47)	0.581
A allele	225	27.85	36	21.18			100	29.24	616	36.84		
*MTHFR* rs3753584 T>C												
TT	312	77.23	69	81.18	1.00		119	69.59	660	78.95	1.00	
CT	88	21.78	15	17.65	1.19 (0.62–2.26)	0.600	51	29.82	165	19.74	**1.78 (1.22–2.60)**	**0.003**
CC	4	0.99	1	1.18	0.69 (0.07–6.69)	0.748	1	0.58	11	1.32	0.50 (0.06–3.95)	0.512
CT+CC	92	22.77	16	18.82	1.15 (0.62–2.16)	0.655	52	30.41	176	21.05	**1.70 (1.17–2.46)**	**0.005**
TT+CT	400	99.01	84	98.82	1.00		170	99.42	825	98.68	1.00	
CC	4	0.99	1	1.18	0.67 (0.07–6.43)	0.725	1	0.58	11	1.32	0.44 (0.06–3.42)	0.428
C allele	96	11.88	17	10.00			53	15.50	187	11.18		
*MTHFR* rs4845882 G>A												
GG	240	59.41	48	56.47	1.00		89	52.05	514	61.48	1.00	
GA	148	36.63	33	38.82	0.82 (0.49–1.37)	0.448	74	43.27	287	34.33	**1.50 (1.06–2.11)**	**0.022**
AA	16	3.96	4	4.71	0.71 (0.21–2.43)	0.586	8	4.68	35	4.19	1.40 (0.62–3.15)	0.415
GA+AA	164	40.59	37	43.53	0.81 (0.49–1.33)	0.402	82	47.95	322	38.52	**1.49 (1.06–2.08)**	**0.021**
GG+GA	388	96.04	81	95.29	1.00		163	95.32	801	95.81	1.00	
AA	16	3.96	4	4.71	0.77 (0.23–2.58)	0.670	8	4.68	35	4.19	1.19 (0.54–2.64)	0.668
A allele	180	22.28	41	24.12			90	26.32	357	21.35		
*MTHFR* rs4846048 A>G												
AA	330	81.68	66	77.65	1.00		135	78.95	694	83.01	1.00	
AG	71	17.57	18	21.18	0.76 (0.41–1.42)	0.391	36	21.05	129	15.43	1.45 (0.95–2.20)	0.082
GG	3	0.74	1	1.18	0.65 (0.05–7.75)	0.729	0	0.00	13	1.55	-	-
AG+GG	74	18.32	19	22.35	0.76 (0.41–1.39)	0.368	36	21.05	142	16.99	1.31 (0.87–1.99)	0.197
AA+AG	401	99.26	84	98.82	1.00		171	100.00	823	98.45	1.00	
GG	3	0.74	1	1.18	0.68 (0.06–8.14)	0.761	0	0.00	13	1.55	-	-
G allele	77	9.53	20	11.76			36	10.53	155	9.27		
*MTHFR* rs9651118 T>C												
TT	135	33.42	22	25.88	1.00		81	47.37	318	38.04	1.00	
TC	199	49.26	48	56.47	0.59 (0.33–1.06)	0.078	68	39.77	418	50.00	**0.66 (0.46–0.94)**	**0.021**
CC	70	17.33	15	17.65	0.72 (0.34–1.54)	0.394	22	12.87	100	11.96	0.88 (0.52–1.50)	0.638
TC+CC	269	66.58	63	74.12	0.62 (0.36–1.09)	0.095	90	52.63	518	61.96	**0.70 (0.50–0.98)**	**0.037**
TT+TC	334	82.67	70	82.35	1.00		149	87.13	736	88.04	1.00	
CC	70	17.33	15	17.65	1.00 (0.52–1.92)	0.995	22	12.87	100	11.96	1.09 (0.66–1.80)	0.727
C allele	339	41.96	78	45.88			112	32.75	618	36.96		

1 Adjusted for age, sex, smoking, status of chronic HBV infection and drinking. Bold values are statistically significant (*P*<0.05).

### MTHFR haplotypes

We constructed six haplotypes of *MTHFR* gene (Table 5). Haplotype analysis of this gene suggested that *MTHFR* A_rs1801133_T_rs3753584_G_rs4845882_A_rs4846048_T_rs9651118_ haplotype was a protective factor for HCC (*P***=**0.008). However, *MTHFR* G_rs1801133_T_rs3753584_G_rs4845882_A_rs4846048_T_rs9651118_ was associated the risk of HCC (*P***=**0.003).

**Table 5:** *MTHFR* haplotype frequencies (%) and risk of HCC

Haplotypes	HCC Cases (*n*=1151)		Controls (*n*=1841)		Crude OR (95% CI)	*P*
	*n*	%	*n*	%		
G_rs1801133_T_rs3753584_G_rs4845882_A_rs4846048_C_rs9651118_	438	38.05	685	37.21	1.00	
A_rs1801133_T_rs3753584_G_rs4845882_A_rs4846048_T_rs9651118_	317	27.54	633	34.38	**0.78 (0.65–0.94)**	**0.008**
G_rs1801133_C_rs3753584_A_rs4845882_A_rs4846048_T_rs9651118_	140	12.63	194	10.54	1.13 (0.88**–**1.45)	0.339
G_rs1801133_T_rs3753584_G_rs4845882_A_rs4846048_T_rs9651118_	111	9.64	112	6.08	**1.55 (1.16–2.07)**	**0.003**
G_rs1801133_T_rs3753584_A_rs4845882_G_rs4846048_T_rs9651118_	109	9.47	170	9.23	1.00 (0.77**–**1.31)	0.984
G_rs1801133_T_rs3753584_A_rs4845882_A_rs4846048_T_rs9651118_	13	1.13	22	1.20	0.92 (0.46**–**1.85)	0.824
Others	23	2.00	25	1.36	1.44 (0.81**–**2.57)	0.216

Bold values are statistically significant (*P*<0.05).

### The power of the present study (α = 0.05)

The present study’s power was determined (α = 0.05). For *MTHFR* rs1801133 polymorphism, the power value was 0.835 in the GA vs. GG genetic model and 0.726 in GA/AA vs. GG genetic model. For *MTHFR* rs3753584 locus, the power of the present study was 0.984 in the CT vs. TT genetic model and 0.965 in CT/CC vs. TT genetic model. When we focused on the *MTHFR* rs965118 polymorphism, the power value was 0.935 in the CT vs. TT genetic model and 0.910 in CT/CC vs. TT genetic model. In addition, the power of *MTHFR* A_rs1801133_T_rs3753584_G_rs4845882_A_rs4846048_T_rs9651118_ and *MTHFR* G_rs1801133_T_rs3753584_G_rs4845882_A_rs4846048_T_rs9651118_ haplotypes were 0.771 and 0.844, respectively.

In the subgroup without chronic HBV infection, we found that the power value was 0.946 in GA vs. GG genetic model and 0.944 in GA/AA vs. GG genetic model for *MTHFR* rs1801133 polymorphism and 0.852 in the CT vs. TT genetic model and 0.803 in CT/CC vs. TT genetic model for *MTHFR* rs3753584 locus. The power value of other subgroups was less than 0.8 (data were not shown).

## Discussion

The HCC susceptibility to individuals may be affected by certain environmental risk factors [27,28]. The high HCC morbidity in certain regions of sub-Saharan Africa and Asia largely attributes to the prevalence of chronic HBV infection. However, individual’s hereditary factor also could influence the risk of HCC [28,29]. MTHFR and 5-MTHF may be implicated in DNA methylation, synthesis and repair. Thus, variants in *MTHFR* could influence the risk of cancer. Several case–control studies were designed to identify the association of *MTHFR* variants with HCC risk. However, the included participants in these studies were relatively small. In addition, the observations of pooled analyses were conflicting [30–33]. Here, we conducted a study with related large sample sizes to assess a potential correlation between *MTHFR* SNPs and susceptibility of HCC. Our results suggested the associations of *MTHFR* rs3753584, rs9651118 and rs1801133 polymorphisms with HCC development. In addition, haplotype analysis of *MTHFR* gene suggested that *MTHFR* G_rs1801133_T_rs3753584_G_rs4845882_A_rs4846048_T_rs9651118_ increased the susceptibility of HCC. The power value of the present study also conferred these associations (power value > 0.80).

Rs1801133 polymorphism is the most extensively studied SNP in *MTHFR* gene. This SNP is a missense variant (Ala→Val at 226 position). MTHFR is vital enzyme in the process of remethylation, and catalyzes Hcy to Met. Rs1801133 locus codes the NH_2_-terminal catalytic domain of MTHFR. MTHFR rs1801133 A allele decreases the activity of protein enzyme [34]. A few case–control studies identified that rs1801133 increased the susceptibility of HCC [15,35,36]. Another study identified that this SNP did not confer risk to HCC [37]. However, Jiao et al. [11] reported that AA genotype and A allele of *MTHFR* rs1801133 may confer a protective effect on HCC in HBV-infected individuals. Some pooled analysis investigated a potential correlation of *MTHFR* rs1801133 with HCC risk. Several meta-analyses reported that *MTHFR* rs1801133 A allele might increase the risk of HCC [31–33,38]. However, in another meta-analysis, Qin et al. [30] suggested that there was no significant association between *MTHFR* rs1801133 locus and HCC risk. The observations were controversial. Thus, we conducted a related large sample size study to investigate the correlation of rs1801133 locus with HCC risk. We concluded that rs1801133 A allele was a protective factor for HCC. Recently, some pooled-analyses demonstrated that this locus is protective for the development of colorectal cancer in Asians [39,40]. An Ala→Val substitute at 226 position in MTHFR may increase the 5,10-methylenetetrahydrofolate for DNA synthesis [41,42], which may be protective for cancer development. In the future, more studies should be conducted to identify whether G→A variant in *MTHFR* rs1801133 locus is a protective factor for HCC development.

To our knowledge, we first clarified the impact of *MTHFR* rs3753584 T>C polymorphism with hepatocarcinogenesis. *MTHFR* rs3753584 is located in nearGene-5, which may regulate the stability, transcription and translation of RNA. Liu et al. [43] suggested that *MTHFR* rs3753584 locus affected the development of lung cancer. Another study found that *MTHFR* rs3753584 variants increased the susceptibility of colon cancer [44]. Here, we identified that *MTHFR* rs3753584 may confer a risk to HCC. Our observation was similar to those findings mentioned above.

Lu et al. [45] conducted a study to detect the correlation of *MTHFR* rs9651118 with susceptibility to breast cancer (BC), and the results suggested that rs9651118 CC genotype decreased the risk of BC. Additionally, in Caucasians, Swartz et al. [46] reported that this variant might be a factor that decreased the susceptibility of lung cancer. In Asians, Ding et al. [47] reported that *MTHFR* rs9651118 T>C polymorphism has a tendency to decrease risk of esophagogastric junction adenocarcinoma. In the present study, we first explored the association between *MTHFR* rs9651118 T>C polymorphism and risk of HCC. Our findings clarified that *MTHFR* rs9651118 C allele was relevant to a protective role for hepatocarcinogenesis. *MTHFR* rs9651118 was an intron SNP, which may influence the alternative splicing pattern. A functional study indicated that rs9651118 CC genotype of *MTHFR*, compared with TT genotype, reduced the Hcy level [48]. Recently, a dose–response meta-analysis concluded that each 5 μmol/l Hcy level promoting increased the incidence of digestive tract cancer by 7% [49]. Thus, *MTHFR* rs9651118 C allele may reduce the Hcy level, and then decrease the susceptibility of HCC.

Our findings suggested *MTHFR* G_rs1801133_T_rs3753584_G_rs4845882_A_rs4846048_T_rs9651118_ increased the susceptibility of HCC. We first investigated the potential correlation of these *MTHFR* tagging SNPs haplotypes with HCC susceptibility. It could be used as a potential biomarker for HCC diagnosis. Previous investigations have focused on the relationship between *MTHFR* haplotypes of these tagging SNPs and cancer susceptibility; however, *MTHFR* G_rs1801133_T_rs3753584_G_rs4845882_A_rs4846048_T_rs9651118_ was not found to be associated with the risk of non-small cell lung cancer [50] and esophagogastric junction adenocarcinoma [47]. In the future, these findings should be further validated.

This hospital-based study might have some potential limitations. First, though we recruited 1507 subjects to investigate a relationship of *MTHFR* tagging SNPs and the risk of HCC here, the sample size might be insufficient to identify weak associations of HCC. Second, in the present study, we only included several risk factors (e.g. sex, chronic HBV infection, age, smoking and drinking), other environmental factors were not considered. Third, the present study was hospital-based, which could not fully represent the Chinese population and the bias might have happened. In the future, population-based investigations are needed to further explore the role of *MTHFR* SNPs to risk of HCC. Fourth, the intake of folate and diet habits were not collected in our study. Thus, we did not focus on the association of *MTHFR* variants and folate level with the susceptibility of HCC. Finally, we only evaluated the *MTHFR* SNPs with HCC, polymorphisms in other one-carbon metabolism genes were not included.

Taken together, in Chinese Han population, *MTHFR* rs9651118 and rs1801133 polymorphisms may be protective for HCC. However, *MTHFR* rs3753584 polymorphism is a candidate for susceptibility to HCC. In the future, these findings should be further validated in additional studies.

## Abbreviations

BC: breast cancer
BCLC: Barcelona Clinic Liver Cancer
CI: confidence interval
HBV: hepatitis B virus
HCC: hepatocellular carcinoma
Hcy: homocysteine
HWE: Hardy–Weinberg equilibrium
LC: liver cancer
MTHFR: methylenetetrahydrofolate reductase
OR: odds ratio
SD: standard deviation
SNP: single nucleotide polymorphism
5-MTHF: 5-methyltetrahydrofolate
